# Endocrine therapy and related issues in hormone receptor-positive early breast cancer: a roundtable discussion by the breast cancer therapy expert group (BCTEG)

**DOI:** 10.1007/s10549-018-4662-9

**Published:** 2018-01-19

**Authors:** Jame Abraham, Humberto Caldera, Robert Coleman, Anthony Elias, Matthew P. Goetz, Muaiad Kittaneh, Elyse Lower, Reshma Mahtani, E. Terry Mamounas, Mark Pegram, Hope Rugo, Lee Schwartzberg, Tiffany Traina, Chuck Vogel

**Affiliations:** 1 Cleveland Clinic, Cleveland, USA; 2 Hematology Oncology Associates, Lake Worth, FL USA; 3 University of Sheffield, Sheffield, UK; 4 University of Colorado, Denver, CO USA; 5 Mayo Clinic, Rochester, USA; 6 Loyola University, Maywood, USA; 7 University of Cincinnati, Cincinnati, USA; 8 University of Miami, Deerfield Beach, FL USA; 9 University of Florida, Orlando, FL USA; 10 Stanford University, Stanford, USA; 11 University of California, San Francisco, USA; 12 University of Tennessee, Knoxville, USA; 13 Memorial Sloan Kettering, New York City, USA

**Keywords:** Breast cancer therapy, Expert group, Endocrine therapy, Extended adjuvant therapy, Genomic testing, Bisphosphonate therapy

## Abstract

**Purpose:**

Management of breast cancer is a rapidly evolving field, and, although evidence-based guidelines are available for clinicians to provide direction on critical issues in patient care, clinicians often left to address these issues in the context of community practice situations with their patients. These include the patient’s comorbid conditions, actual versus perceived benefit of treatments, patient’s compliance as well as financial/reimbursement issues, and long-term tolerability of therapy.

**Methods:**

A meeting of global oncology experts was convened in January 2017 with the belief that there is a gap in clinical practice guidance on several fundamental issues in breast cancer care, particularly in the community setting, where oncologists may encounter multiple tumor types. The goal was to discuss some of the most important questions in this area and provide some guidance for practicing oncologists.

**Results:**

Topics addressed included risk of contralateral breast cancer recurrence in patients with estrogen receptor-positive early breast cancer who have undergone 5 years of adjuvant endocrine therapy, adverse events associated with endocrine therapy and their management, emergent data on adjuvant bisphosphonate therapy and its apparent benefit in reducing breast cancer recurrence, recent findings of extended adjuvant endocrine therapy trials, and the use of currently available genomic biomarker tests as a means of further informing treatment decisions.

**Conclusions:**

A summary of the discussion on these topics and several ‘expert opinion statements’ are provided herein in an effort to convey the collective insights of the panel as it relates to current standard practice.

## Introduction: about the breast cancer therapy expert group (BCTEG) panel

Management of breast cancer is a dynamic and constantly evolving field of oncology. Clinical guidance statements, recommendations, and meta-analyses are available for clinicians that provide direction on issues relating to the use of adjuvant endocrine therapy, and extended adjuvant (EA) endocrine therapy in patients with estrogen receptor-positive (ER+), early breast cancer (EBC). Despite these resources, however, many issues surrounding the use of, for example, EA endocrine therapy, remain unresolved, and clinicians are left to address critical questions of care with their patients in the context of in-practice clinical considerations, including comorbidities, patient’s compliance, actual versus perceived benefit of therapies, patient age, financial/reimbursement issues, and tolerability of therapies over the long term.

The breast cancer therapy expert group (BCTEG) panel is composed of expert physicians and clinical researchers, all of whom have dedicated their careers to the treatment of patients with breast cancer and have published extensively on the topics in question. The purpose of convening the panel was to discuss important developments related to breast cancer management, with a particular emphasis on new findings and/or areas where guidance from established bodies, such as the National Comprehensive Cancer Network (NCCN) and the American Society for Clinical Oncology (ASCO) may be unresolved or less established. The goal is to elicit the group’s opinions on the topic as it relates to their own clinical practices and, more importantly, how this might impact those practicing in the community setting, where multiple tumor types, other than breast cancer, are frequently encountered. Importantly, this article is not intended to replace any existing guidance or to be an exhaustive review of the topic. Rather, it is intended to present a concise synopsis of the relevant data in this area and summarize the consensus opinion of the expert group.

## Meeting objectives and role of funding sources

A BCTEG meeting was convened in January 2017 with the goal of conducting an informal roundtable discussion on some of the most important topics related to the use of endocrine therapy in the treatment of hormone receptor-positive EBC. Topics discussed included issues surrounding the role of contralateral prophylactic mastectomy (CPM) in ER+ EBC, adherence to endocrine therapies, and the use of multi-gene assays, which can provide prognostic information and guide decisions on systemic adjuvant therapy [1–5]. Emergent data regarding the benefits of using adjuvant bisphosphonates on recurrence and survival outcomes in postmenopausal women, as reported by the Early Breast Cancer Trialist’s Collaborative Group (EBCTCG) [6], were also discussed, as were studies presented at the 2016 San Antonio Breast Cancer Symposium (SABCS), which raised questions regarding the benefit of using EA endocrine therapy with aromatase inhibitors (AIs) beyond 5 years [7–9]. This article is intended to provide a collective summary of the participants’ expert opinions on current standard practice relating to these issues. An unrestricted educational grant for this activity was provided by Biotheranostics, Inc., with additional funding provided by Total Health Conferencing, a medical education company. The faculty were compensated for their participation, and topics of discussion were selected by the faculty and by Total Health Conferencing. It is recognized that many of the panelists may have relationships with corporate entities, both related and unrelated to the topics in question; content of the discussions, and any expert opinions presented herein, was intended to be based on the panelists’ own expert clinical experience and insight, and is understood not to be influenced by any corporate relationship or interest.

## Segment 1: contralateral breast cancer risk

### Background

This segment of the discussion was focused on risk reduction and contralateral breast cancer events in women with established unilateral breast cancer (without risk for hereditary breast or ovarian cancer syndromes) and was aimed at eliciting the expert panel’s opinion on estimated annual risk of a patient developing a metachronous contralateral breast cancer (CBC) from diagnosis, and after receiving 5 years of endocrine therapy. Related to this discussion was their current assessment of available strategies and/or therapeutic options to reduce CBC risk [i.e., surveillance, contralateral prophylactic mastectomy (CPM), adjuvant hormonal therapy], and whether an individual patient’s risk of CBC influenced their recommendations regarding the use of EA hormonal therapy.

## Discussion

The panel agreed that, based on results from the Oxford overview, adjuvant endocrine therapy confers a substantial benefit in terms of reducing the risk of CBC for women with ER+ disease, and that older estimates of CBC recurrence risk at 10–15 years (~ 5 to 8%) are not reflective of what is seen today, especially for women with ER+ disease who have received 5 years of endocrine therapy; as most recently gleaned from the NSABP B-42 trial, this risk is approximately 3% [7]. Strategies to reduce CBC risk include surveillance for a woman with an intact contralateral breast, and mammography remains the cornerstone of surveillance. American Society for Clinical Oncology (ASCO) guidelines recommend mammography once per year, and there is no recommendation for MRI or additional functional imaging for patients with average risk [5]. Thus, for women with ER+ disease taking hormonal therapy, after 5 years, one can confidently say that the risk of CBC at least over the next 10 years is quite low, and the use of CPM in these patients would be largely unnecessary. Given the low risk of a CBC in years 5 through 10, it was generally agreed that the risk of CBC should not weigh heavily into treatment decisions, and that discussions for extending adjuvant therapy, at least at present, should focus mainly on the risk of distant recurrence. In terms of surveillance, panelists were in agreement with ASCO guidelines, which recommend yearly mammography, with the understanding that more frequent screening (i.e., every 6 months) may cause undue anxiety for patients and increase costs, and the acknowledgement that studies (e.g., MAMMO50) evaluating less frequent schedules have completed accrual, and will be reported in the next few years [5, 10].

*Expert Opinion Statement 1* The use of EA endocrine therapy in the context of reducing contralateral breast cancer should be highly individualized, and be based on age, as well as relevant clinicopathological factors and tolerability. Contralateral prophylactic mastectomy is considered largely unnecessary in patients with standard risk (i.e., patients without history of hereditary breast cancer syndromes), given the very low risk of contralateral breast cancer, particularly in ER+ patients who have received 5 years of initial adjuvant endocrine therapy.

*Expert Opinion Statement 2* Many patients may be currently over-screened with every 6-month mammography of the affected breast, which can increase patient anxiety and costs. Therefore, the panel recommends that the current ASCO guidelines for yearly mammographic screening be followed.

## Segment 2: adverse events and patient compliance

### Background

This segment of the discussion was focused on adverse event management and patient compliance with endocrine therapy, with a specific focus on AIs, and was aimed at eliciting the expert panel’s perception of the incidence and impact of adverse events (AEs) on compliance. Related to this discussion was the potential impact of patient adherence on treatment outcome, and strategies recommended by the panel to manage specific endocrine therapy-associated AEs such as arthralgia, hot flashes, vaginal dryness or atrophy, and bone loss.

## Discussion

The panelists discussed the definition of adherence and compliance. The term “adherence” could be considered as a composite of compliance (i.e., how well the drug regimen is followed) and persistence (i.e., how long the patient follows the treatment) [2, 11]. In this regard, it was also noted that reliable measures of adherence are, for the most part, currently lacking. It was agreed that there is considerable discordance between the perception of adherence by most clinicians and nonadherence rates as reported in clinical trials, in clinical practice, and in medication refill record databases [12, 13]. It was acknowledged that lack of adherence is associated with poorer survival in the EA setting [14], and that several factors could impact adherence, including the physicians themselves (i.e., failure to provide a prescription), and patient factors (i.e., failure to fill the prescription if given) [15]. AEs, including arthralgia, hot flashes, and vaginal dryness or atrophy, are also extremely important concerns for patients on AIs. Management strategies discussed included education on life style changes, such as exercise, weight reduction, stress management, and improving sleep habits. Analgesics were also agreed to be effective for the treatment of AI-induced arthralgia. The panel also noted vaginal dryness as an underreported and poorly managed side effect of anti-estrogen therapy, which must be addressed and managed. It was suggested that topical nonhormonal vaginal moisturizers and lubricants may be used to improve symptoms of vaginal dryness, but persistent gynecologic symptoms may necessitate a discussion regarding topical estrogen therapy. In this regard, the panel noted that, in some studies, significant increases in plasma estrogen have been observed with topical estrogen use and that concerns over their use in women with breast cancer are common [16]. Importantly, however, the panel noted that to date no studies have reported an increased risk of breast cancer recurrence in patients receiving vaginal estrogens, nor has a critical estradiol level been defined that is associated with higher rates of recurrence in AI-treated women. Nevertheless, it was agreed that the use of topical estrogens should be based on physician–patient discussions regarding individual risk and benefit.

Regarding the issue of bone health, the panel recommended periodic evaluation of bone densitometry and management of vitamin D deficiency in order to minimize bone loss. Depending on bone loss risk factors including personal or family history of fracture, smoking, and alcohol usage, along with bone densitometry measurements, the use of bisphosphonates or denosumab may be indicated [17]. The selective serotonin reuptake inhibitors, including duloxetine and venlafaxine, can also be considered for symptoms such as musculoskeletal pain and hot flashes [18, 19]. In tamoxifen-treated women, antidepressants with little or no inhibition of CYP2D6 should be preferred over antidepressants with potent CYP2D6 inhibition activity, as these medications may result in decreased conversion of tamoxifen to the active metabolite, endoxifen. Lastly, changing from one aromatase inhibitor to another or to a selective estrogen receptor modulator (i.e., tamoxifen) is another option to improve hormone therapy tolerance [20]. The panel agreed that many women gather information through internet-based resources (including social media) regarding the potential AEs associated with AIs and tamoxifen, and that internet bias may not provide an accurate risk versus benefit assessment for patients. Patient education regarding the benefits (and risks) of endocrine therapy is therefore essential to minimize any uncertainties or misinformation.

*Expert Opinion Statement 1* Education to improve patient’s perception of adjuvant endocrine therapy benefit and understanding of side effects is a mainstay of improving adherence to endocrine therapies.

*Expert Opinion Statement 2* Management strategies to address side effects related to endocrine therapy should include exercise (moderate-intensity physical activity for 30 min/day, 5 times/week), correcting and maintaining vitamin D levels in accordance with current bone health guidelines, and use of nonhormonal or, if necessary, hormonal topical agents to address vaginal dryness and atrophy; analgesics and antidepressants may also be considered for arthralgia and hot flashes.

## Segment 3: adjuvant bisphosphonates and RANK ligand inhibitors

### Background

This segment of the discussion was focused on eliciting the expert panel’s opinions on the use of adjuvant bisphosphonate and RANK ligand (RANK-L) inhibitor therapy for reducing risk of breast cancer recurrence. Specifically, the panel sought to identify potential candidates for these treatments and to evaluate side effect/risk profiles of both therapies, and their impact on treatment choices. A related line of discussion was whether the decision to offer patients adjuvant bisphosphonate or RANK-L inhibitor therapy influenced the recommendation to also offer EA hormonal therapy.

## Discussion

The panel discussion was centered on the results of the Oxford meta-analysis, which included nearly 19,000 women, approximately 12,000 of whom were postmenopausal [6]. In the postmenopausal subset of breast cancer patients, bisphosphonate use (of any type, oral or intravenous), prescribed shortly after diagnosis, was associated with highly significant reductions in all recurrences as well as bone recurrences, and in breast cancer mortality, whereas no such benefit was seen in the premenopausal subset [6]. The panel acknowledged the limitations of applying the results of this meta-analysis in terms of obtaining regulatory approval for these agents in postmenopausal women. They also acknowledged the comparatively smaller dataset for the RANK-L inhibitor denosumab, which was felt at present, to be too immature to show a clear benefit in reducing recurrence or improving survival. It was suggested, however, that denosumab may be more appropriately used to reduce fracture risk, particularly in the EA setting, as the FDA already approved denosumab for the treatment of AI-induced bone loss [21, 22]. It was agreed by the panel that, in Europe, adjuvant bisphosphonates would typically be used in women with intermediate to high risk, assuming that they are either postmenopausal or on ovarian suppression. Duration of treatment in this setting is unclear, but most participants used a schedule of every 6 months zoledronic acid (4 mg intravenous) for 3 years. By comparison, in the United States, the panel agreed that the use of bisphosphonates in the adjuvant setting for the purpose of reducing recurrence is limited by regulatory obstacles. Bisphosphonates are not presently FDA approved or covered by insurance for this indication and currently their use is approved only for those patients who have continued bone loss after receiving first-line oral bisphosphonates (i.e., alendronate). There are no data to suggest intervention with a bone-targeted agent in the context of EA therapy will reduce late recurrence, and therefore the decision to offer a bone-targeted therapy was not though the influence the decision to offer EA therapy.

*Expert Opinion Statement 1* Setting aside issues with regulatory approval and payers/reimbursement, given evidence for a benefit of adjuvant bisphosphonates in reducing recurrence, distant recurrence, and improving survival in postmenopausal women receiving adjuvant endocrine therapy, adjuvant bisphosphonates can be considered for women with early-stage breast cancer.

*Expert Opinion Statement 2* Notwithstanding a lack of demonstrated survival benefit, a RANK-L inhibitor, denosumab, may be considered for patients receiving adjuvant endocrine therapy in view of its approved indication for preventing bone loss in patients receiving AIs.

## Segment 4: current perspectives on the use of multi-gene assays and duration of endocrine therapy

This segment of the discussion was focused on the current guidelines regarding the use of multi-gene assays and was aimed at eliciting the expert panel’s opinion on the optimal duration of adjuvant endocrine therapy for patients with hormone receptor-positive early breast cancer and, specifically, whether there is a clinically meaningful benefit of extending AI-based endocrine therapy to 10 years for selected patients. Related to this discussion was the panel’s current recommendation for stratifying patients for EA endocrine therapy (i.e., do they currently use clinicopathologic features with or without genomic assays for this purpose).

## Discussion

The panel discussion was centered on the new guidelines on multi-gene assays from ASCO, which were noted to have deviated somewhat from the initial guidance on this topic, and some inconsistencies between these guidelines and those of the National Comprehensive Cancer Network (NCCN) were also noted (e.g., for node-positive patients). The panel noted the absence of emergent data from key trials, particularly MINDACT [23] in the 2016 ASCO guidance on biomarkers [3], and suggested that, due to the rapid advances in this field, updates in this guidance should occur more frequently. The panel agreed that multi-gene assays do provide important information, which can be useful when communicating recurrence risk and benefits of therapy with the patients. For example, if chemotherapy can reduce recurrence risk by 30%, one could safely recommend the treatment if recurrence risk was ~ 50%; by comparison, if recurrence risk was only 10–15% for a given patient, the small absolute benefit from chemotherapy would certainly be outweighed by the risk of intervention [3, 24]. There was agreement that further validation studies are needed and that tumor biology (as revealed by multi-gene assays) must also be considered in the continuum of other clinicopathologic factors.

In view of recently reported results from SABCS, participants agreed that the benefit of EA endocrine therapy is, overall, modest, and some participants cited the need for a meta-analysis of all EA endocrine studies that could provide further clarification on the issue. For example, as reported in the NSABP-42 study, disease-free survival at ~ 7 years was 84.7 and 81.3% for patients on extended adjuvant AI (letrozole) and placebo groups, respectively (3.4% absolute benefit), and the difference did not technically reach statistical significance, due to adjustments for interim analyses (hazard ratio = 0.85, *P* = 0.048; *P* value for significance set at 0.0418) [7]. In view of these and other results, there is a need to more effectively identify patients most likely to benefit from EA endocrine treatment. To this end, the panel recognized the availability of several genomic biomarker tests which, in conjunction with clinicopathologic factors, may provide additional prognostic information on recurrence risk; these include Oncotype Dx, Breast Cancer Index (BCI), PAM 50 Risk of Recurrence (ROR), EndoPredict, and MammaPrint [3]. All of these tests can be useful to identify patient subsets that have an extremely low risk of recurrence. The PAM 50 ROR score, for example, has been shown to provide additional prognostic information over and above standard clinical factors on risk of distant recurrence in the ABCSG 8 trial [25], and Level 1 evidence supporting the use of the 70-gene signature (MammaPrint) to further inform decisions on avoiding the use of adjuvant chemotherapy has also recently been published from the MINDACT trial [23]. Many of the participants reported using the 21-gene Oncotype Dx Recurrence Score (RS) to help inform their decisions on the use of adjuvant chemotherapy in node-positive (1–3 lymph nodes) and node-negative ER+ patients, in the context of other clinicopathologic factors. While again citing the need for additional validation, at least one of these tests, BCI, has also been shown to predict response to endocrine therapy, and many participants reported having used BCI, at least on occasion (or more frequently) to further inform their treatment decisions for patients whose need for an additional 5 years of endocrine therapy was less certain (most notably, node-negative patients) [26–28]. It was also acknowledged that major cancer staging manuals, such as the American Joint Commission on Cancer (AJCC) forthcoming 8th Edition, have already incorporated these multi-gene panels into their risk assessment models.

*Expert Opinion Statement 1* Although further validation studies are needed, the use of multi-gene assays may provide important additional information that can guide treatment decisions; this information should be considered in the context of other clinicopathologic factors.

*Expert Opinion Statement 2* Recently reported results from EA endocrine therapy trials suggest an overall modest benefit of extending endocrine therapy beyond 5 years; the currently available genomic biomarker assays may be useful to further inform treatment decisions in patients where uncertainty may exist (e.g., node-negative and/or poor tolerability).

## Conclusion

In the face of emergent clinical trial data, optimal treatment for patients with early breast cancer will continue to evolve. The panel recognized the limitations of current guidance surrounding the use of EA endocrine therapy for patients with ER+ disease, and the importance of future discussions on this topic. They also recognize the importance of forthcoming data that will further inform treatment decisions in this area. Until such data become available, however, the panel recommends a highly individualized approach, with shared patient–physician decision-making, and a strong emphasis on patient education to help improve adherence and persistence when selecting patients for CPM, EA endocrine therapy, and/or adjuvant bisphosphonate treatment.

## Note added in proof

Shortly after this expert roundtable meeting was convened, a guideline statement was published by a joint Cancer Care Ontario (CCO)/ASCO working group and expert panel, which offers recommendations on the use of adjuvant bisphosphonates in postmenopausal women undergoing systemic adjuvant therapy for breast cancer. (http://ascopubs.org/doi/full/10.1200/JCO.2016.70.7257) The reader is referred to this statement for further guidance and clarification on this issue. In addition, the authors note the recently published focused update of the ASCO guidance on the use of biomarkers which details the results from MINDACT supporting the use of MammaPrint to inform decisions on avoiding the use of systemic adjuvant chemotherapy in women with high-clinical risk and low-genomic risk early-stage invasive ER+ breast cancer. (http://ascopubs.org/doi/pdf/10.1200/JCO.2017.74.0472).
